# Effect of HbA1c and Inflammatory Hematological Markers on Postoperative Outcomes in Diabetic Patients Undergoing Major Abdominal Surgery

**DOI:** 10.7759/cureus.100900

**Published:** 2026-01-06

**Authors:** Mahnoor, Talha Rao, Madina Riaz, Renad Al Mefleh, Zahra Liaqat Ali, Hania Noor, Afifah Mehmood, Razwan Ashraf, Syed A Hussain

**Affiliations:** 1 Intensive Care Unit, St George’s University Hospital, London, GBR; 2 Medicine, Tehsil Headquarter Hospital, Mailsi, PAK; 3 Internal Medicine, Bolan Medical College, Quetta, PAK; 4 Acute Medicine, Queen Elizabeth Hospital, Birmingham, GBR; 5 Pediatrics, Jordanian Royal Medical Services, Amman, JOR; 6 Medicine, D.G. (Dera Ghazi) Khan Medical College, Dera Ghazi Khan, PAK; 7 Medicine, Multan Medical and Dental College, Multan, PAK; 8 Internal Medicine, District Headquarter Hospital Rawalpindi, Rawalpindi, PAK; 9 General Surgery, Federal Government Polyclinic (FGPC) Hospital, Islamabad, PAK; 10 Medicine, Aziz Bhatti Shaheed Teaching Hospital, Gujrat, PAK; 11 Surgical B Ward, Ayub Teaching Hospital, Abbottabad, PAK

**Keywords:** abdominal surgery, hba1c, hematological markers, nlr, postoperative complications

## Abstract

Background

Diabetic patients undergoing major abdominal surgery are at increased risk of postoperative complications due to chronic hyperglycemia and altered inflammatory responses. Glycated hemoglobin (HbA1c) reflects long-term glycemic control, while complete blood count-derived inflammatory markers provide insight into systemic inflammation. This study evaluated the association of HbA1c and inflammatory hematological markers with postoperative outcomes in diabetic patients undergoing major abdominal surgery.

Methods

This prospective observational cohort study was conducted at Nawaz Sharif Medical College and Aziz Bhatti Shaheed Teaching Hospital, Gujrat, Pakistan, over a 12-month period from February 2024 to January 2025. Adult diabetic patients undergoing elective or emergency major abdominal surgery were enrolled using consecutive sampling. Preoperative HbA1c and inflammatory hematological parameters, including neutrophil-to-lymphocyte ratio (NLR), platelet-to-lymphocyte ratio (PLR), and lymphocyte-to-monocyte ratio (LMR), were assessed. Postoperative complications occurring during hospitalization or within 30 days were recorded. Multivariable logistic regression was performed to identify independent predictors of postoperative complications.

Results

A total of 205 patients were included in the final analysis. The mean age was 58.6 ± 9.8 years, and 57.6% were male patients. Overall, 77 patients (37.6%) developed postoperative complications. Patients with complications had significantly higher mean HbA1c levels compared with those without complications (8.8 ± 1.3% vs. 7.6 ± 1.2%, p < 0.001). Inflammatory markers, including total leukocyte count, neutrophil count, NLR, and PLR, were significantly elevated, whereas LMR was significantly lower in the complication group (all p < 0.001). On multivariable analysis, HbA1c (adjusted OR 1.42, 95% CI 1.18-1.72), NLR (adjusted OR 1.31, 95% CI 1.18-1.46), and PLR (adjusted OR 1.08, 95% CI 1.03-1.14) were independently associated with postoperative complications.

Conclusion

Elevated HbA1c and inflammatory hematological markers are independently associated with adverse postoperative outcomes in diabetic patients undergoing major abdominal surgery. These readily available parameters may aid in preoperative risk stratification and perioperative optimization.

## Introduction

Diabetes mellitus is a common comorbidity among patients undergoing major abdominal surgery and is associated with increased postoperative morbidity and mortality compared with non-diabetic individuals [1]. Impaired wound healing, increased susceptibility to infection, and dysregulated inflammatory responses contribute to poor surgical outcomes in this population [2]. Given the growing burden of diabetes worldwide, identifying simple and reliable preoperative predictors of postoperative complications remains a key concern in perioperative care.

Glycated hemoglobin (HbA1c) is an established indicator of long-term glycemic control and reflects cumulative hyperglycemia over the preceding two to three months. Elevated HbA1c levels have been linked to a higher risk of postoperative complications, including surgical-site infections, sepsis, prolonged hospital stay, and mortality [3,4]. Unlike perioperative blood glucose measurements, which may fluctuate due to stress and acute illness, HbA1c provides a stable marker of chronic metabolic status and has therefore gained importance in preoperative risk assessment [5,6]. A recent meta-analysis demonstrated a significant association between poor preoperative glycemic control and adverse surgical outcomes across multiple surgical disciplines [7].

In addition to chronic hyperglycemia, systemic inflammation plays a pivotal role in postoperative recovery. Diabetes is characterized by a persistent low-grade inflammatory state that may be further amplified by surgical stress [8]. Hematological parameters obtained from routine complete blood counts, such as neutrophil, lymphocyte, and platelet counts, have emerged as practical indicators of systemic inflammation. Derived inflammatory indices, including the neutrophil-to-lymphocyte ratio (NLR), platelet-to-lymphocyte ratio (PLR), and lymphocyte-to-monocyte ratio (LMR), have shown prognostic value in predicting postoperative complications and infectious outcomes in various surgical settings [9-12].

Evidence from observational studies and meta-analyses suggests that elevated NLR and PLR are associated with increased postoperative morbidity, while lower LMR reflects impaired immune regulation and poorer outcomes [10]. These markers are inexpensive, readily available, and require no specialized assays, making them particularly suitable for use in routine clinical practice, especially in resource-limited settings. However, most previous studies have evaluated glycemic control or inflammatory markers independently, and data examining their combined effect on postoperative outcomes in diabetic patients undergoing major abdominal surgery remain limited [11,13,14]. Furthermore, regional data from South Asian populations are scarce. This study was designed to determine the independent association of HbA1c and complete blood count-derived inflammatory indices with postoperative complications after adjusting for relevant clinical and surgical confounders.

## Materials and methods

This prospective observational cohort study was conducted at Nawaz Sharif Medical College in collaboration with Aziz Bhatti Shaheed Teaching Hospital, Gujrat, Pakistan. The study duration was 12 months, commencing in February 2024 and concluding in January 2025. The study was designed and reported in accordance with the Strengthening the Reporting of Observational Studies in Epidemiology (STROBE) guidelines for observational research to ensure methodological rigor and transparency.

The study population comprised adult patients with a confirmed diagnosis of diabetes mellitus who underwent major abdominal surgery during the study period. Major abdominal surgery was defined as any elective or emergency intra-abdominal surgical procedure performed under general anesthesia with an expected operative duration exceeding 2 hours and requiring postoperative hospitalization of at least 48 hours, including but not limited to colorectal, hepatobiliary, gastric, pancreatic, and major intestinal surgeries. Eligible participants were adults aged 18 years and above with either type 1 or type 2 diabetes mellitus, diagnosed at least three months prior to surgery, and with a documented preoperative HbA1c measurement performed within four weeks before the surgical procedure. Patients were required to provide written informed consent prior to enrollment.

Patients were excluded if they had preexisting hematological disorders such as leukemia, lymphoma, aplastic anemia, or hemoglobinopathies; active malignancy receiving chemotherapy or radiotherapy; chronic inflammatory or autoimmune diseases; end-stage renal disease on dialysis; chronic liver disease with decompensation; acute infection or sepsis at the time of surgery; recent blood transfusion within four weeks prior to surgery; or if they were receiving systemic corticosteroids or immunosuppressive therapy. Patients who died intraoperatively or within 24 hours of surgery due to causes unrelated to metabolic or inflammatory status were also excluded to avoid outcome misclassification.

The sample size was calculated using standard epidemiological methods for cohort studies. Based on previously published regional and international literature, the expected rate of postoperative complications in diabetic patients with poor glycemic control was assumed to be approximately 35%, compared with 20% in those with acceptable glycemic control. With a confidence level of 95%, a study power of 80%, and a two-sided alpha of 0.05, the minimum required sample size was calculated to be 186 patients. To account for potential attrition, incomplete records, and missing laboratory data, a 10% contingency was added, resulting in a final target sample size of approximately 205 patients. Consecutive eligible patients presenting during the study period were enrolled until the required sample size was achieved, using a non-probability consecutive sampling technique. Consecutive sampling was deliberately chosen to minimize selection bias by ensuring systematic inclusion of all eligible patients presenting in routine clinical practice, including both elective and emergency surgical cases, rather than selectively recruiting participants based on investigator discretion. This approach is commonly used and considered appropriate in hospital-based observational cohort studies where random sampling is impractical due to the unpredictable nature of surgical admissions, particularly in emergency settings.

Baseline demographic and clinical data were collected preoperatively through structured medical record review and patient interviews. Variables included age, sex, body mass index, duration and type of diabetes mellitus, comorbid conditions such as hypertension, ischemic heart disease, and chronic kidney disease, smoking status, American Society of Anesthesiologists (ASA) physical status classification, indication for surgery, type of surgical procedure, and whether the surgery was elective or emergency. These variables were considered potential confounders due to their known association with postoperative outcomes and inflammatory status.

Preoperative glycemic control was assessed using HbA1c, which was measured within four weeks prior to surgery using routine hospital laboratory methods based on high-performance liquid chromatography standardized to the National Glycohemoglobin Standardization Program. HbA1c values were analyzed both as a continuous variable and as categorical variables using clinically relevant cutoffs to stratify glycemic control. Fasting and random blood glucose levels measured perioperatively were recorded but were not used as primary exposure variables to avoid short-term glycemic variability bias.

Inflammatory hematological markers were assessed using routine complete blood count parameters obtained within 24 hours prior to surgery. These included total leukocyte count, neutrophil count, lymphocyte count, platelet count, hemoglobin level, and hematocrit. Derived inflammatory indices were calculated using standard formulas, including the NLR, PLR, and LMR. These indices were selected because they are cost-effective, routinely available, and do not require proprietary instruments or licensed scoring systems.

Postoperative outcomes were prospectively recorded from the time of surgery until hospital discharge or up to 30 postoperative days, whichever occurred first. The primary outcome was the occurrence of postoperative complications, defined as any deviation from the normal postoperative course. Complications were categorized into surgical-site infections, wound dehiscence, postoperative sepsis, pneumonia, urinary tract infection, anastomotic leak, postoperative ileus, acute kidney injury, cardiovascular events, and need for reoperation. Complications were graded according to standardized clinical definitions documented in hospital records. Secondary outcomes included length of postoperative hospital stay, admission to the intensive care unit, and in-hospital mortality.

Data were collected using a predefined case record form by trained investigators and cross-verified with hospital electronic and paper records to ensure accuracy. To minimize information bias, laboratory values were extracted directly from the hospital laboratory information system, and outcome assessment was performed by clinicians not involved in the laboratory analysis.

Potential confounders were identified a priori based on clinical relevance and previous literature and were adjusted for during statistical analysis. These included age, sex, body mass index, duration of diabetes, comorbidities, ASA class, type and urgency of surgery, and baseline hemoglobin levels. Although several clinically relevant confounders were identified a priori and adjusted for during statistical analysis, certain variables could not be systematically measured or standardized across all patients. These included perioperative insulin management protocols, timing and choice of prophylactic antibiotics, preoperative nutritional status, and individual surgeon experience. These factors were therefore not included in the multivariable models and are acknowledged as potential sources of residual confounding. Missing data were assessed for pattern and proportion. If missing data for any variable exceeded 5%, multiple imputation using chained equations was planned under the assumption of data missing at random. Variables with less than 5% missing data were handled using complete-case analysis.

Statistical analysis was performed using a standard statistical software package SPSS version 28 (IBM Corp., Armonk, NY). Continuous variables were assessed for normality using the Shapiro-Wilk test. Continuous variables were assessed for normality. Variables with normal distribution were expressed as mean ± standard deviation, while non-normally distributed variables were summarized as median with interquartile range. Comparisons between groups were performed using the independent-samples t-test and the chi-square test. Multivariable logistic regression analysis was used to determine the independent association of HbA1c and inflammatory hematological markers with postoperative complications after adjusting for confounders. Postoperative complications were analyzed as a binary outcome, and severity grading was not performed, which may limit differentiation between minor and major complications. Adjusted odds ratios with 95% confidence intervals were reported. A p-value of less than 0.05 was considered statistically significant.

Ethical approval for the study was obtained from the Ethical Review Committee of Nawaz Sharif Medical College and Aziz Bhatti Shaheed Teaching Hospital, Gujrat, Pakistan (Reference No. ERC/NMC-HI-ERC/24/12). The study was conducted in accordance with the Declaration of Helsinki. Written informed consent was obtained from all participants prior to enrollment, and confidentiality of patient data was strictly maintained throughout the study by anonymizing records and restricting data access to the research team only.

## Results

A total of 212 diabetic patients undergoing major abdominal surgery were initially assessed for eligibility during the study period. Seven patients were excluded due to incomplete preoperative laboratory data (n = 4) or withdrawal of consent (n = 3). The final analysis, therefore, included 205 patients, yielding a data completeness rate of 96.6%. No systematic pattern of missing data was observed, and complete-case analysis was applied.

The mean age of the study population was 58.6 ± 9.8 years, with a male predominance (n = 118, 57.6%). The majority of patients had type 2 diabetes mellitus (91.2%), with a median diabetes duration of eight years (IQR: 5-12). Elective procedures accounted for 72.7% of surgeries, while 27.3% were performed on an emergency basis. There were no statistically significant differences in baseline demographic variables between patients who developed postoperative complications and those who did not, except for ASA physical status, duration of diabetes, and emergency surgery status. Baseline demographic and clinical characteristics are summarized in Table 1. The mean preoperative HbA1c of the cohort was 8.1 ± 1.4%. Patients who developed postoperative complications had significantly higher HbA1c levels compared with those without complications (8.8 ± 1.3% vs. 7.6 ± 1.2%, p < 0.001). Similarly, total leukocyte count, neutrophil count, and derived inflammatory indices, including NLR and PLR, were significantly elevated in the complication group. Preoperative laboratory parameters are detailed in Table 2.

**Table 1:** Baseline Demographic and Clinical Characteristics of the Study Population (n = 205) Baseline demographic and clinical characteristics of diabetic patients undergoing major abdominal surgery according to postoperative complication status. Data are presented as mean ± SD, median (IQR), or n (%). Independent-samples t-test, Mann-Whitney U test, and χ² test were applied as appropriate. BMI = body mass index; ASA = American Society of Anesthesiologists.

Variable	Total (n = 205)	No Complications (n = 128)	Complications (n = 77)	Test Statistic	p-Value
Age (years, mean ± SD)	58.6 ± 9.8	57.9 ± 9.4	59.8 ± 10.3	t = 1.32	0.189
Male, n (%)	118 (57.6)	71 (55.5)	47 (61.0)	χ² = 0.57	0.450
BMI (kg/m², mean ± SD)	27.1 ± 4.2	26.8 ± 4.0	27.6 ± 4.5	t = 1.29	0.199
Duration of diabetes (years, median (IQR))	8 (5-12)	7 (4-10)	10 (6-14)	U = 3821	0.004
Type 2 diabetes, n (%)	187 (91.2)	118 (92.2)	69 (89.6)	χ² = 0.36	0.548
Hypertension, n (%)	132 (64.4)	78 (60.9)	54 (70.1)	χ² = 1.77	0.183
ASA class III-IV, n (%)	96 (46.8)	48 (37.5)	48 (62.3)	χ² = 12.1	0.001
Emergency surgery, n (%)	56 (27.3)	24 (18.8)	32 (41.6)	χ² = 13.0	<0.001

**Table 2:** Preoperative Glycemic and Inflammatory Hematological Parameters Preoperative HbA1c and inflammatory hematological parameters stratified by postoperative complications. Data are expressed as mean ± SD or median (IQR). Group comparisons were performed using t-test or Mann-Whitney U test. HbA1c = glycated hemoglobin; NLR = neutrophil-to-lymphocyte ratio; PLR = platelet-to-lymphocyte ratio; LMR = lymphocyte-to-monocyte ratio.

Parameter	No Complications (n = 128)	Complications (n = 77)	Test Statistic	p-Value
HbA1c (%), mean ± SD	7.6 ± 1.2	8.8 ± 1.3	t = 6.47	<0.001
Hemoglobin (g/dL), mean ± SD	12.4 ± 1.5	11.6 ± 1.6	t = 3.61	<0.001
Total leukocyte count (×10⁹/L), mean ± SD	7.8 ± 2.1	10.2 ± 2.8	t = 6.34	<0.001
Neutrophil count (×10⁹/L), mean ± SD	4.9 ± 1.8	7.1 ± 2.3	t = 7.01	<0.001
Lymphocyte count (×10⁹/L), mean ± SD	1.9 ± 0.6	1.4 ± 0.5	t = 5.41	<0.001
Platelet count (×10⁹/L), mean ± SD	258 ± 72	302 ± 81	t = 3.89	<0.001
NLR, median (IQR)	2.6 (1.9-3.4)	5.1 (3.9-6.8)	U = 2014	<0.001
PLR, median (IQR)	128 (104-156)	198 (162-241)	U = 1897	<0.001
LMR, median (IQR)	4.1 (3.3-5.0)	2.6 (2.1-3.4)	U = 2134	<0.001

Patients who developed postoperative complications had significantly higher HbA1c levels compared with those without complications (8.8 ± 1.3% vs. 7.6 ± 1.2%, p < 0.001). Similarly, total leukocyte count, neutrophil count, and derived inflammatory indices, including NLR and PLR, were significantly elevated in the complication group. Preoperative laboratory parameters are detailed in Table 2.

Overall, 77 patients (37.6%) developed at least one postoperative complication. Surgical-site infection was the most frequent complication, followed by postoperative sepsis and pneumonia. Patients with poor glycemic control (HbA1c ≥ 8.0%) had a significantly higher rate of complications compared with those with HbA1c <8.0% (51.9% vs. 25.4%, p < 0.001). Postoperative outcomes are summarized in Table 3.

**Table 3:** Postoperative Outcomes of the Study Population Postoperative outcomes in diabetic patients undergoing major abdominal surgery. Data are presented as n (%) or median (IQR). ICU = intensive care unit.

Outcome	Frequency, n (%)
Any postoperative complication	77 (37.6)
Surgical-site infection	39 (19.0)
Postoperative sepsis	28 (13.7)
Pneumonia	22 (10.7)
Acute kidney injury	17 (8.3)
Anastomotic leak	11 (5.4)
Cardiovascular events	9 (4.4)
ICU admission	46 (22.4)
In-hospital mortality	8 (3.9)
Length of hospital stay (days), median (IQR)	9 (6-14)

On univariable analysis, elevated HbA1c, higher NLR, higher PLR, lower LMR, emergency surgery, and ASA class III-IV were significantly associated with postoperative complications. Multivariable logistic regression analysis demonstrated that HbA1c, NLR, and PLR remained independently associated with postoperative complications after adjustment for confounders. The results of the multivariable analysis are presented in Table 4.

**Table 4:** Multivariable Logistic Regression Analysis for Predictors of Postoperative Complications Multivariable logistic regression analysis of factors associated with postoperative complications. Results are reported as adjusted ORs with 95% CIs. A p-value <0.05 was considered statistically significant. OR = odds ratio; CI = confidence interval; HbA1c = glycated hemoglobin; NLR = neutrophil-to-lymphocyte ratio; PLR = platelet-to-lymphocyte ratio; LMR = lymphocyte-to-monocyte ratio; ASA = American Society of Anesthesiologists.

Variable	Adjusted OR	95% CI	p-Value
HbA1c (per 1% increase)	1.42	1.18-1.72	<0.001
NLR (per unit increase)	1.31	1.18-1.46	<0.001
PLR (per 10-unit increase)	1.08	1.03-1.14	0.002
LMR	0.74	0.61-0.89	0.001
ASA class III-IV	2.11	1.18-3.77	0.011
Emergency surgery	2.36	1.29-4.32	0.005
Duration of diabetes (years)	1.04	1.01-1.08	0.018

## Discussion

This prospective observational study evaluated the impact of preoperative glycemic control and inflammatory hematological markers on postoperative outcomes in diabetic patients undergoing major abdominal surgery. The findings demonstrate that elevated HbA1c and heightened systemic inflammatory response, reflected by increased NLR and PLR and reduced LMR, are significantly associated with adverse postoperative outcomes. Importantly, HbA1c, NLR, and PLR remained independent predictors of postoperative complications after adjustment for clinically relevant confounders, highlighting the combined prognostic value of chronic hyperglycemia and preoperative inflammatory status.

The baseline characteristics of the study population are consistent with the epidemiological profile of surgical diabetic cohorts reported in previous studies, with a predominance of older male patients and type 2 diabetes mellitus [15]. Age, sex, and BMI did not differ significantly between patients with and without postoperative complications, suggesting that metabolic and inflammatory factors may exert a stronger influence on postoperative risk than demographic variables alone. However, a longer duration of diabetes was significantly associated with complications, which aligns with earlier evidence linking chronic diabetes to cumulative microvascular and immune dysfunction, thereby increasing surgical vulnerability [16,17].

The significant association between higher ASA physical status (III-IV) and postoperative complications observed in this study corroborates existing literature identifying ASA classification as a robust predictor of perioperative morbidity [17]. Similarly, emergency surgery was independently associated with adverse outcomes, reflecting limited preoperative optimization, higher physiological stress, and an amplified inflammatory response, findings that are consistently reported in surgical outcome studies involving diabetic patients [18].

One of the key findings of this study is the strong association between elevated preoperative HbA1c levels and postoperative complications. Patients who developed complications had significantly higher HbA1c values, and each 1% increase in HbA1c independently increased the odds of postoperative complications by 42%. This finding is in agreement with multiple observational studies and meta-analyses that have demonstrated poor long-term glycemic control as a predictor of surgical-site infections, sepsis, prolonged hospital stay, and mortality [19,20].

Chronic hyperglycemia impairs neutrophil chemotaxis, phagocytosis, and oxidative burst activity, while also promoting endothelial dysfunction and impaired collagen synthesis, thereby delaying wound healing and increasing susceptibility to infection [21,22]. The significantly higher rate of complications observed among patients with HbA1c ≥8.0% in the present study supports the growing consensus that HbA1c should be incorporated into preoperative risk stratification rather than relying solely on perioperative glucose levels [3].

In addition to glycemic control, this study highlights the prognostic significance of inflammatory hematological markers. Patients with postoperative complications demonstrated significantly higher total leukocyte and neutrophil counts, along with lower lymphocyte counts, reflecting a shift toward a pro-inflammatory and immunosuppressed state. These findings are consistent with previous studies demonstrating that preoperative leukocytosis and neutrophilia are associated with increased postoperative infectious and non-infectious complications [23].

Derived inflammatory indices further strengthened these observations. The NLR was markedly elevated in patients with complications and independently predicted adverse outcomes in multivariable analysis. Similar associations have been reported across a range of surgical specialties, including colorectal, hepatobiliary, and gastrointestinal surgery, where elevated NLR has been linked to postoperative infections, sepsis, and prolonged hospitalization [10,24].

The PLR also emerged as an independent predictor of postoperative complications in the present study. Elevated PLR likely reflects both platelet-mediated inflammatory activation and relative lymphopenia, which together contribute to impaired immune regulation and endothelial dysfunction [25]. Previous studies have demonstrated similar associations between elevated PLR and postoperative morbidity, particularly in patients with diabetes and cardiovascular comorbidities [26,27]. In contrast, a lower LMR was protective, supporting prior evidence that preserved lymphocyte counts and lower monocyte-driven inflammation are associated with improved postoperative recovery [28,29].

Taken together, the results of this study emphasize that postoperative outcomes in diabetic patients are influenced not only by operative factors but also by the interplay between chronic glycemic exposure and preoperative inflammatory status. Unlike costly or proprietary biomarkers, HbA1c and complete blood count-derived inflammatory indices are widely available, inexpensive, and require no special equipment or licensing, making them particularly suitable for routine risk assessment in resource-limited settings.

The findings of this study support the integration of HbA1c and simple inflammatory hematological markers into preoperative risk stratification for diabetic patients undergoing major abdominal surgery. Identification of patients with poor glycemic control and heightened inflammatory status may allow targeted perioperative optimization, closer postoperative monitoring, and early intervention to reduce complication rates. Given their low cost, wide availability, and ease of interpretation, these markers offer a practical and scalable approach to improving surgical outcomes, particularly in high-volume and resource-constrained healthcare settings.

This study has certain limitations that merit consideration. Its single-center design may limit generalizability, and although multiple confounders were adjusted for, residual confounding cannot be entirely excluded. Additionally, inflammatory markers were measured only preoperatively, and dynamic postoperative changes were not assessed. Nevertheless, the prospective design, standardized data collection, and comprehensive multivariable analysis strengthen the validity of the findings.

## Conclusions

This study demonstrates that elevated HbA1c and inflammatory hematological markers, particularly NLR and PLR, are independently associated with adverse postoperative outcomes in diabetic patients undergoing major abdominal surgery. Incorporating these readily available parameters into preoperative evaluation may improve risk stratification, guide perioperative optimization, and ultimately enhance surgical outcomes in this high-risk population.
